# The integration of psychosocial care, ethical governance, and patient-centered research in current and future approaches in the field of vascularized composite allotransplantation

**DOI:** 10.3389/frtra.2026.1869486

**Published:** 2026-05-25

**Authors:** Martin Kumnig, Lisa Hopfgartner, Gerald Brandacher

**Affiliations:** 1 Department of Psychiatry, Psychotherapy, Psychosomatics and Medical Psychology, Center for Advanced Psychology in Plastic and Transplant Surgery, Medical University of Innsbruck, Innsbruck, Austria; 2 Department of Visceral, Transplant and Thoracic Surgery, Medical University of Innsbruck, Innsbruck, Austria

**Keywords:** artificial intelligence, bioethics, identity, normothermic machine perfusion, psychosocial outcomes, tissue engineering, trust, vascularized composite allotransplantation (VCA)

## Abstract

Vascularized composite allotransplantation (VCA) has transitioned from an experimental endeavor to a clinically viable option for patients with complex tissue loss. While advances in microsurgery, immunosuppression, and perioperative care have improved graft survival, long-term success remains contingent upon psychosocial integration and robust ethical governance. These developments are reshaping not only clinical outcomes but also the conceptual foundations of transplantation. VCA uniquely challenges traditional paradigms, as it is primarily life-enhancing rather than life-saving, thereby intensifying ethical scrutiny regarding risk–benefit proportionality. Psychosocial factors such as identity reconstruction, body wholeness, personal autonomy, adherence, and long-term mental health are central determinants of graft success. Concurrently, innovations such as AI-driven allocation systems, big data analytics, and digital monitoring introduce novel ethical concerns related to autonomy, transparency, data governance, and equity. A key objective of this mini-review is to critically examine how emerging technological innovations in VCA intersect with psychosocial processes and bioethical principles, and to outline a framework for their responsible integration into clinical practice. It argues that technological progress must be embedded within a patient-centered approach that prioritizes lived experience, relational ethics, and trust. The integration of person-centered care models, longitudinal assessments, and interdisciplinary “team science” approaches is essential for ensuring that innovation translates into meaningful, equitable, and ethically sustainable improvements in patient outcomes Ultimately, the future of VCA depends on aligning biomedical innovation with psychosocial resilience and ethical legitimacy, ensuring that reconstructive success translates into meaningful improvements in quality of life and providing access to these transplants for all patients in need.

## 1 Introduction: toward a biopsychosocial-technological framework in VCA

Vascularized composite allotransplantation (VCA) is one of the most complex areas of transplantation because it intersects surgical innovation, identity restoration, and ethical decision-making. VCA directly addresses basic aspects of selfhood, embodiment, and social interaction as a reconstructive technique involving visible and functionally integrated bodily parts, such as the face or upper extremities, going well beyond technical restoration (1, 2).

Over the past decade, the field has entered a phase of rapid technological acceleration. Advances in organ preservation and normothermic machine perfusion, immunomodulatory strategies aimed at immunosuppression minimization and tolerance induction, advanced immune monitoring, robotic-assisted microsurgery, and digital innovations such as artificial intelligence (AI) and big data analytics are reshaping the boundaries of what is technically feasible (3–7). In parallel, emerging fields including regenerative medicine, tissue engineering, and xenotransplantation are expanding the conceptual and biological limits of transplantation, offering potential solutions to donor scarcity while simultaneously introducing new layers of ethical and psychosocial complexity (8, 9).

Despite these advances, a critical gap persists: technological progress has outpaced the systematic integration of psychosocial and bioethical frameworks into clinical practice. In VCA, the graft is not only functional but also visible, identity-relevant, and socially embedded. As such, successful outcomes depend on processes of psychological embodiment, identity reconstruction, and sustained adherence, all of which are shaped by individual, relational, and societal factors (1, 9). In addition, new ethical issues pertaining to distributive justice, informed consent, autonomy, and data governance are brought about by evolving technologies, especially when it comes to AI-driven decision-making and increasingly intricate preservation and allocation systems (6, 10).

These developments underscore the necessity of a biopsychosocial-technological paradigm that systematically integrates ethical governance, psychosocial treatment, and biomedical innovation. By evaluating transplantation success in terms of lived experience, identity integration, and trust—both at the level of the individual patient and within the larger social context—such a methodology goes beyond conventional outcome measurements (1, 11). Importantly, this approach is in line with the increasing focus on interdisciplinary “team science” and person-centered care precision medicine, acknowledging that sustained advancement in VCA necessitates the coordinated involvement of surgical, scientific, psychological, ethical, and technological expertise (11).

This mini-review builds on this framework to examine how emerging innovations in transplantation medicine reshape psychosocial processes and ethical considerations in VCA, and to outline key directions for their responsible and patient-centered integration into clinical practice.

## 2 The psychosocial architecture of VCA in the context of innovation

VCA engages fundamental dimensions of identity, embodiment, and social reintegration in ways that are unparalleled in other areas of transplantation medicine. The psychosocial context of transplantation is changing dramatically due to the quick development and clinical application of new transplantation technologies Because transplanted structures such as the face or upper extremities are both functionally and symbolically integrated into the self, recipients are required to incorporate a graft that is neither fully “self” nor entirely “other.” This process has been conceptualized as the emergence of a “third identity,” reflecting a dynamic interplay between pre-injury self, donor characteristics, and post-transplant adaptation (1, 9). Therefore, research promotes for careful consideration of patients’ emotional well-being and coping capacities before and after any VCA procedure (12).

At a neurobiological level, embodiment is supported by neuroplastic processes that facilitate the incorporation of the graft into the body schema. However, this integration is not guaranteed and may remain incomplete or unstable. Psychological rejection—e.g., face transplant recipients may report distress related to unfamiliar appearance, while upper extremity recipients may struggle to integrate a transplanted hand into daily activities despite favorable functional outcomes—can occur independently of immunological rejection or functional regain and has been associated with impaired adherence and adverse clinical outcomes (9, 11).

Psychosocial adaptation in VCA is further shaped by pre-existing psychiatric conditions, including depression, anxiety, and post-traumatic stress disorder. Rather than representing static contraindications, these factors interact dynamically with the demands of transplantation. Chronic stress contributes to increased allostatic load, which may negatively affect immune function, adherence, and long-term recovery (e.g., this may clinically manifest in patients with prior trauma histories who struggle with rehabilitation adherence) (12). Consequently, contemporary approaches emphasize psychosocial optimization, including pre-habilitation, structured caregiver involvement, and longitudinal support.

Importantly, the psychosocial architecture of VCA is increasingly mediated by technological innovation. Digital surgical planning, advanced visualization techniques, and evolving monitoring systems shape patient expectations, influence self-perception, and structure postoperative adaptation. As a result, psychosocial processes in VCA must be understood as embedded within a technologically mediated clinical environment, where subjective experience and technical intervention are closely intertwined.

## 3 Technological innovation as a driver of psychosocial and ethical complexity

Technological innovation is redefining VCA by expanding surgical possibilities while creating new psychosocial and ethical challenges (e.g., surgical manipulation can be perceived as a violation of Islamic bodily ethics). Each major innovation introduces specific tensions that extend beyond technical considerations and directly affect patients, families, and healthcare systems (13).

While these developments enhance clinical feasibility, they also challenge foundational ethical constructs by blurring the boundaries between life and death (14). For instance, prolonged ex-vivo perfusion of a donor limb may improve graft viability but may also be perceived by donor families as a form of “technological prolongation”. These conceptual shifts may influence donor family perceptions and complicate processes of meaning-making and trust (3, 13, 15).

Advances in immunomodulation and regenerative medicine, such as immunosuppression minimization and bioengineered tissues, could reduce lifelong immunosuppression. This clinical progress changes the psychosocial aspects of transplantation, disrupting coping and identity as patients shift toward functional normalization. Additionally, using bioengineered or synthetic tissues raises concerns about authenticity, bodily integrity, and human boundaries (5), with synthetic grafts often viewed as less natural than donor tissue.

The increasing use of AI and big data analytics introduces additional layers of complexity. Importantly, these technologies also offer meaningful clinical benefits, including improved donor-recipient matching, earlier rejection prediction, optimization of immunosuppressive regimens, and reduced clinician workload through automated monitoring systems. For example, AI-supported imaging analysis may help identify early vascular compromise before overt graft failure occurs. These systems may improve donor-recipient matching and rejection prediction, but also raise concerns regarding bias, transparency, and explainability (6, 10). From a psychosocial perspective, reliance on algorithmic systems may alter perceptions of agency and control, particularly if clinical decisions are experienced as externally determined. This affects informed consent, patient autonomy, and trust—key factors for patient participation and integrating artificial organ technologies in clinical settings. Specifically, this includes the tension between the patient's hope for a cure and the experimental nature of the trial, as well as the question of equitable access to these high-tech treatments (16).

Digital health technologies, including remote monitoring systems and patient-reported outcome measures (PROMs), further extend the reach of transplantation into patients' everyday lives. For example, remote wound monitoring may reduce travel burden and enable earlier intervention, while also contributing to surveillance-related distress. While these tools enable continuous assessment and early detection of complications, they may also contribute to a sense of constant surveillance, reduced autonomy, and “digital fatigue” (17). Concerns regarding data privacy, ownership, and security are increasingly salient, particularly in the context of large-scale data integration. Moreover, disparities in digital literacy and access risk exacerbating existing inequalities in transplantation care.

Xenotransplantation requires clarification in the context of VCA, as there is currently no clinical precedent for xenogeneic hand, face, or other composite tissue transplantation in humans. Its relevance remains primarily preclinical and conceptual. Advances in genetically modified porcine donors and xenogeneic tolerance strategies may eventually inform solutions to donor scarcity in VCA, although major barriers remain—particularly the high immunogenicity of skin-containing grafts (8, 18–20). Future psychosocial concerns may include identity integration, stigma, and acceptance of animal-derived tissues, while ethical concerns include zoonotic surveillance, informed consent, and public health oversight.

Across these domains, technological innovation emerges as a double-edged process: it simultaneously expands therapeutic possibilities and generates new forms of vulnerability. Psychosocial and ethical challenges are therefore not peripheral but intrinsic to the development and implementation of innovative transplantation technologies.

## 4 Toward an integrated biopsychosocial-technological model

The increasing complexity of VCA necessitates a shift toward an integrated biopsychosocial-technological model, in which clinical outcomes are understood as the result of interacting biological, psychological, social, and technological processes (1, 9). Within this framework, success is no longer defined solely by graft survival or functional restoration but includes identity integration, sustained adherence, and meaningful social participation.

In this context, the integration of technological innovation must be accompanied by deliberate efforts to sustain relational continuity and ethical clarity. Trust is essential for patient engagement and adherence, yet it is increasingly challenged by the complexity and opacity of emerging technologies. Maintaining trust requires transparency, effective communication, and the preservation of human oversight within technologically mediated systems (6, 10). In this context, the integration of technological innovation must be accompanied by deliberate efforts to sustain relational continuity and ethical clarity.

The complexity of VCA further underscores the importance of interdisciplinary collaboration. Effective programs depend on the coordinated involvement of surgery, psychiatry, psychology, ethics, immunology, rehabilitation medicine, and data science. Such “team science” approaches enable the integration of diverse perspectives and support comprehensive, patient-centered care (21, 22). Beyond clinical practice, interdisciplinary collaboration is also essential for addressing the broader ethical and societal implications of emerging technologies.

A further priority within this framework is the development of longitudinal approaches to psychosocial assessment. Processes such as identity reconstruction, embodiment, and adaptation evolve over time and require continuous evaluation. The integration of quantitative measures, including PROMs, with qualitative methodologies allows for a more nuanced understanding of patient trajectories (17, 23). Digital technologies offer new opportunities for real-time data collection but must be carefully balanced against concerns related to surveillance burden, privacy, and inequity (24).

Finally, the biopsychosocial-technological model implies a shift toward anticipatory ethics. Rather than addressing ethical challenges retrospectively, this approach emphasizes the proactive integration of psychosocial and ethical considerations into the development and implementation of new technologies. Such an orientation is essential for ensuring that innovation in VCA remains aligned with core principles of autonomy, justice, and respect for persons.

In sum, this model provides a conceptual framework for aligning technological advancement with human experience. It offers a pathway toward sustainable, equitable, and patient-centered progress in VCA.

## 5 Future directions: translating innovation into psychosocially and ethically sustainable practice

The rapid expansion of transplantation medicine and VCA needs a forward-thinking framework that incorporates psychosocial and bioethical concerns in technology advancement. Table 1 applies the biopsychosocial-technological framework by linking key arguments to clinical domains, showing how each technology brings unique psychosocial and ethical impacts on patient outcomes.

**Table 1:** Psychosocial and bioethical challenges of emerging innovations in vascularized composite allotransplantation (VCA).

Technological innovations	Psychosocial challenges	Bioethical challenges
Normothermic Machine Perfusion (NMP)	Potential destabilization of donor family trust; perception of “technological reanimation” impacting the grieving process	Concerns regarding bodily integrity
Artificial Intelligence (AI) in Allocation & Monitoring	Perceived loss of human agency; fear of the dehumanization of clinical decision-making and patient-physician relationship	Algorithmic bias leading to healthcare inequities; lack of transparency in “black box” predictive modeling
Xenotransplantation (currently in preclinical in VCA)	Species-identity crisis; potential social stigma, alienation, and psychological rejection of the “non-human” graft	Zoonotic risk management; mandatory lifelong surveillance vs. the individual's right to privacy and liberty
Bio-engineered/Synthetic Tissues	Perceptions of “unnaturalness”; challenges in the psychological incorporation of synthetic grafts into the body schema	Therapeutic misconception in early-phase trials; balancing long-term safety profiles against the pressure for innovation
Robotic Surgery and Virtual Surgical Planning (VSP)	Risk of unrealistic expectations and “perfectionism” due to highly optimized pre-operative 3D simulations	Ensuring the validity of informed consent in highly complex, technologically mediated surgical interventions
Targeted Immunomodulation (e.g., Treg recruitment)	Paradoxical identity distress as patients transition from a “chronic patient” status to a perceived “healthy” state	Ethical proportionality and justification for application in pediatric populations for non-life-saving procedures
Digital PROMs & Remote Monitoring	Digital burden and “surveillance anxiety”; the feeling of perpetual clinical observation within the private sphere	Data security and privacy; risk of a “digital divide” exacerbating inequalities in access to high-end transplant care

### 5.1 From technological innovation to psychosocial risk stratification

One of Table 1's main conclusions is that psychosocial risk heterogeneity is directly correlated with technical complexity. Innovations such as machine perfusion, cryopreservation, AI-based allocation, and xenotransplantation could change patients' social, emotional, and cognitive interactions with transplantation in addition to changing clinical procedures.

Future research should focus on creating psychosocial risk profiles unique to technology, incorporating factors like:

- perceived bodily integrity and identity coherence
- tolerance for uncertainty and technological mediation
- trust in institutions and algorithmic systems
- capacity for long-term adherence under surveillance conditions

Recent research highlights that psychosocial variables are dynamic trajectories that require long-term monitoring (12). A crucial next step is the creation of adaptable psychosocial phenotyping models, which my be aided by digital PROMs and real-time data collection.

Crucially, reductionism must be avoided in these methods. Digital monitoring has the potential to improve early distress detection and psychological burden, but it also runs the danger of creating “surveillance anxiety” and digital weariness, especially that are already at risk (12). This duality necessitates careful calibration of patient autonomy and monitoring intensity, as seen in Table 1.

### 5.2 Machine perfusion and the redefinition of death and donation

Among the most revolutionary advancements in transplantation medicine are advances in machine perfusion and organ preservation. *Ex vivo* perfusion can considerably increase preservation durations and enhance graft quality, according to recent research, particularly in VCA where ischemia-reperfusion damage remains a major limitation.

However, as indicated in Table 1, these technologies also put fundamental ethical principles to the test. These challenges are no longer theoretical; rather, they are actively influencing clinical practice and policy considerations, as demonstrated by recent discussion in the field.

Future research must therefore address: How donor families perceive machine-mediated preservation and potential “reanimation”? Whether current consent frameworks adequately capture these technological realities? How to maintain public trust in the context of increasingly complex procurement processes? Empirical bioethics studies, which combine qualitative interviews with donor families and experimental vignette designs, will be fundamental for developing ethically sound policies.

### 5.3 AI, big data, and algorithmic governance

With applications ranging from donor-recipient matching to rejection prediction, AI-driven decision-making signifies a paradigm shift in transplantation. These systems bring new types of moral risk and epistemic opacity, but may also improve waitlist prioritization, rejection monitoring, and clinical efficiency.

As seen in Table 1, the main emotional impact of AI goes beyond technological issues. Patients may lose their sense of agency and interpersonal connection, especially if they believe that healthcare decisions are made by algorithms rather than by cooperative negotiation and shared decision making. Recent literature underscores three critical research priorities: (1) explainability and openness of algorithms, (2) using representative datasets to reduce bias, and (3) including human supervision (also known as “human-in-the-loop” models). Moreover, AI systems that have to be trained on past transplant data run the risk of sustaining institutional injustices, which disproportionately impact vulnerable groups. Therefore, distributive faireness and algorithmic justice must be incorporated into bioethical frameworks: including (1) standardized reporting guidelines for AI in transplantation, (2) patient-centered consent models addressing algorithmic involvement, and (3) interdisciplinary oversight committees integrating clinicians, ethicists, and data scientists.

### 5.4 Xenotransplantation and the expansion of identity boundaries

Xenotransplantation is one of the most psychologically and ethically significant innovations in transplantation. Although, it presents a possible remedy for the shortage of organs, it also seriously questions ideas of human identity and physical integrity. These concerns may be amplified by religious beliefs, perceived stigma, or discomfort with animal-derived organs.

Simultaneously, xenotransplantation imposes significant regulatory constraints, such as lifelong infection surveillance and personal autonomy restrictions, which are justified by public health considerations. This poses a unique conflict in transplantation medicine between individual rights and collective safety. Therefore, future studies must examine how recipients perceive “species identity” and embodiment, assess the psychosocial effects of ongoing surveillance procedures, and create moral frameworks that strike a balance between personal freedom and public health obligations. To properly capture these characteristics, interdisciplinary cooperation with disciplines like anthropology, philosophy, and science and technology studies will be crucial.

### 5.5 Regenerative medicine, immunomodulation, and the changing meaning of transplantation

Advances in regenerative medicine and immunological tolerance induction are altering the conceptual boundaries of transplantation. The goal of techniques like bioengineered tissues and cell-based therapies is to lessen or do away with the requirement for lifelong immunosuppression. These advancements could greatly increase safety and expand eligibility requirements form a clinical standpoint. But they also provide fresh psychosocial dilemmas, as Table 1 shows. For instance, the shift from a chronic sickness identification (such as “transplant patients”) to a condition of functional normalcy may upset social roles and coping strategies. Systematic research is needed on this phenome, which is comparable to survivorship transitions in oncology. Additionally, early-phase clinical trials in regenerative medicine raise concerns about therapeutic misconception, particularly in vulnerable patients seeking transformative interventions. The following are some future research priorities:

- longitudinal studies on identity transitions following tolerance induction,
- development of ethical frameworks for first-in-human trials, and
- integration of psychosocial endpoints into regenerative medicine studies.

### 5.6 Toward global equity and inclusive innovation

Table 1 highlights a cross-cutting theme: the risk of technological stratification. Many of the technologies that have been explored, such as AI-systems, machine perfusion platforms, robotic surgery, are resource-intensive and may exacerbate global disparities in access to transplantation. In order to harmonize psychosocial evaluation and bioethical governance across transplant centers, recent evaluations highlight the necessity of international collaboration and standardization (9, 25, 26). Without such initiatives, VCA runs the risk of becoming extremely specialized intervention that is only available in certain high-income environments. Thus, future directions should prioritize the development of scalable, cost-effective transplantation models, including a range of demographic and ethical variables in datasets used for AI training and clinical research.

## 6 Conclusion

A prime example of modern transplantation therapy is VCA, which combines significant psychosocial and ethical complexity with quick technology advancement. As discussed throughout this review, innovative developments in fields are not only expanding surgical capabilities but also radically changing how transplantation is perceived, understood, and regulated.

The preceding sections highlighted that these innovations have a direct impact on key psychosocial processes such as identity reconstruction, embodiment, and long-term adaptation, while also posing new ethical challenges relating to autonomy, trust, transparency, and equity. In this situation, graft survival and functional restoration are no longer sufficient indicators of VCA success. Instead, it must be viewed as a multifaceted result that encompasses meaningful engagement in social life, relationship well-being, and long-term psychological integration.

This review has argued for the adoption of a biopsychosocial-technological model, in which biomedical innovation is systematically integrated with psychosocial care and ethical governance. Within this framework, technological advances are not viewed as isolated achievements but as components of a broader ecosystem that shapes patient experience and clinical outcomes. Central to this model is the recognition that trust, interdisciplinary collaboration, and longitudinal psychosocial assessment are essential for translating innovation into sustainable and patient-centered practice. Hence, the integration of person-centered approaches, patient-reported outcomes, and interdisciplinary “team science” enables a more comprehensive understanding of success—one that is co-constructed by clinicians, researchers, and patients alike.

Ultimately, the future of VCA depends on its capacity to align technological possibility with human experience. Only by embedding innovation within a framework that respects identity, autonomy, and the lived reality of patients can VCA fully realize its transformative potential—not only restoring form and function, but supporting the reconstruction of personhood.
